# UK pneumonectomy outcome study (UKPOS): a prospective observational study of pneumonectomy outcome

**DOI:** 10.1186/1749-8090-4-41

**Published:** 2009-07-30

**Authors:** Ellie S Powell, Adrian C Pearce, David Cook, Paul Davies, Ehab Bishay, Geoffrey MR Bowler, Fang Gao, UKPOS Co-ordinators

**Affiliations:** 1 Heart of England NHS Trust, Birmingham, UK; 2 Guy's and St Thomas' NHS Trust, London, UK; 3 University Hospitals Birmingham NHS Trust, Birmingham, UK; 4 Royal Infirmary Edinburgh, Edinburgh, UK

## Abstract

**Background:**

In order to assess the short term risks of pneumonectomy for lung cancer in contemporary practice a one year prospective observational study of pneumonectomy outcome was made. Current UK practice for pneumonectomy was observed to note patient and treatment factors associated with major complications.

**Methods:**

A multicentre, prospective, observational cohort study was performed. All 35 UK thoracic surgical centres were invited to submit data to the study. All adult patients undergoing pneumonectomy for lung cancer between 1 January and 31 December 2005 were included. Patients undergoing pleuropneumonectomy, extended pneumonectomy, completion pneumonectomy following previous lobectomy and pneumonectomy for benign disease, were excluded from the study.

The main outcome measure was suffering a major complication. Major complications were defined as: death within 30 days of surgery; treated cardiac arrhythmia or hypotension; unplanned intensive care admission; further surgery or inotrope usage.

**Results:**

312 pneumonectomies from 28 participating centres were entered. The major complication incidence was: 30-day mortality 5.4%; treated cardiac arrhythmia 19.9%; unplanned intensive care unit admission 9.3%; further surgery 4.8%; inotrope usage 3.5%. Age, American Society of Anesthesiologists physical status ≥ P3, pre-operative diffusing capacity for carbon monoxide (DLCO) and epidural analgesia were collectively the strongest risk factors for major complications. Major complications prolonged median hospital stay by 2 days.

**Conclusion:**

The 30 day mortality rate was less than 8%, in agreement with the British Thoracic Society guidelines. Pneumonectomy was associated with a high rate of major complications. Age, ASA physical status, DLCO and epidural analgesia appeared collectively most associated with major complications.

## Background

Pneumonectomy is a valuable surgical option for anatomically resectable non-small cell lung cancer when a wedge resection, lobectomy or other lesser resection will not clear the tumour. However, pneumonectomy for lung cancer is associated with significant morbidity and mortality. There have been conflicting findings concerning the important risk factors for poor outcomes following a pneumonectomy.[1-10]

In this prospective, multi-centre observational cohort study of UK pneumonectomy outcome (UKPOS) we examined current UK anaesthetic and surgical practice for pneumonectomy, investigated rates of major complications, in-hospital and 30-day mortality rates, and their risk factors.

## Methods

### Study centres

Having obtained Multi-centre Research Ethics Committee approval, all thoracic centres (n = 35) across the UK were invited to submit pneumonectomy data. The Research and Development Directorates in 28 centres were willing to participate and obtained Site Specific Approval. Each thoracic centre had a Principal Investigator (PI) responsible for collecting and entering the data on an anonymous UKPOS data sheet and submitting it to the Data Centre at Birmingham Heartlands Hospital, England. The PI was responsible for ensuring as many patients were recruited as possible and for following-up the study patients for 30 days.

### Study participants

All adult patients (>18 years of age) undergoing elective pneumonectomy for lung cancer from 1^st ^January to 31^st ^December 2005 were eligible for inclusion in the study. We included those patients for whom pneumonectomy was the intended primary procedure and those who were scheduled for more limited lung resection but had on-table conversions to pneumonectomy. We excluded patients undergoing pleuropneumonectomy, extended pneumonectomy, completion pneumonectomy following previous lobectomy and pneumonectomy for benign disease.

### Data collection

Data were collected prospectively using identical data collection sheets with over 50 essential fields (Additional file 1: Data collection sheet). Accompanying material defined each field to establish conformity amongst the reporting centres. When a patient's data were complete they were transmitted by the PI in anonymous format to the Data Centre and checked for obvious errors. The minimum predicted post-operative (ppo) values for forced expiratory volume in one second (FEV_1_) and carbon monoxide diffusing capacity (DLCO) were calculated using the following formula: preoperative value in percent × number of segments remaining/19 (total). A separate communication contained patient identifiers, where written consent had been obtained, and these data were passed to the Office for National Statistics for long-term follow-up.

### Outcomes after pneumonectomy

For the assessment of outcome, the end point was a patient suffering a major complication. This was defined as any of the following: treated cardiac arrhythmias, unplanned intensive care admissions, further surgery, inotrope usage, or 30 day mortality.

### Statistical analysis

Summary statistics quoted are medians and ranges as many characteristics discussed have skew-distributions. Subgroup comparisons used Mann-Whitney tests or other non-parametric tests. Categorical data were compared by Chi-squared or Fisher exact tests as appropriate. Stepwise logistic regression, as implemented in SPSS 15.0, was used to determine odds ratios and 95% confidence intervals for variables between patients with and without post-operative major complications and to explore the multi-factor association with all observed risk factors.

## Results

### Characteristics of Thoracic Centres and the patients

Four of 28 centres with Site Specific Approval did not perform any pneumonectomies during the study period. The Data Centre received datasheets from 24 UK thoracic surgical centres (range 2–38 pneumonectomies/centre), of which 12 centres (50%) performed more than 10 pneumonectomies in 2005. Data were collected for 312 patients who had elective pneumonectomies for lung cancer from 1^st ^January to 31^st ^December 2005. The Society of Cardiothoracic Surgeons of Great Britain returns for 2005–2006 recorded 416 pneumonectomies for primary lung cancer, but this number would include completion or extended pneumonectomies not relevant to our study.[11] Our data capture of 312 pneumonectomies therefore represents at least 75% of the UK case load in that year. Analysis of variation between centres using the Mantel-Haenszel method revealed that centres were not homogeneous in respect of several surgical and patient characteristics considered here. However having captured such a large part of UK case load, we view this as representing UK practice and only discuss the centres here as a collective.

As shown in Table 1, two-thirds of patients were male, and they were older and heavier smokers than female patients. Out of 310 patients with known American Society of Anesthesiology (ASA) Physical Statuses, the majority (94%) were ≥ ASA P2 (P2-53.1, P3-40.6 and P4-3.5%). Most patients (69%, n = 215) had tumours staged IIb and less and 10.6% (n = 33) patients received neo-adjuvant chemo- or radiotherapy.

**Table 1:** Characteristics of the patients

	Male (n = 209) *	Female (n = 101)*
*Median (range)*		
Age (years)	65 (30–86)	59 (36–80)
Weight (kg)	78 (49–116)	63 (40–112)
Body Mass Index	25.4 (16.6 – 37.0)	25.1 (18.0 – 41.0)
Smoking history (pack-yr)	40 (5–250, n = 172)	35 (4–420, n = 81)
*gender of two cases not recorded		

**Pathological tumour stage**	% (n)	
IA	5.1 (15)	
IB	35.9 (106)	
IIA	4.4 (13)	
IIB	27.5 (81)	
IIIA	21.7 (64)	
IIIB	5.4 (16)	
Total of 295 recorded		

**Pulmonary function tests**	**Number of patients**	**Median (range)**
Forced expiratory volume_1 _(FEV_1_) L	309	2.2 (0.5 – 4.1)
FEV_1_/Forced vital capacity (FVC) %	294	69 (37 – 100)
FEV_1 _% predicted	308	78 (17.3 – 130.8)
Minimum ppo FEV_1 _% predicted	306	39.3 (8.1 – 69.3)
DLCO mmol/min/kPa	153	5.8 (0.8 – 12.6)
DLCO % predicted	163	70 (29 – 136)
Minimum ppo DLCO % predicted	163	35.7 (15.3 – 71.6)
Continuous walking distance on flat without stopping, metre	238	1,500 (4–15,000)

### Preoperative cardiopulmonary function

Table 1 lists the baseline pulmonary function test results of the study group. 261 of 312 (83.7%) patients had pre-operative oxygen saturation on air measured. 235 (75.3%) had oxygen saturations more than 95%, 22 (7.1%) were within 90–94%, 4 (1.3%) were less than 90%, and 51 were unknown (16.3%).

### Intra-operative management

Right-sided operations were performed in 35.3% (n = 110) of patients. Pneumonectomy took a median of 150 (55–600) minutes to complete, and 32.7% (n = 102) of operations were on-table conversions from lobectomy to pneumonectomy. The majority of surgeons used a stapling device to close the bronchial stump (n = 249, 87.1%) compared to a hand sewn suture technique (n = 37, 12.9%). The median of 233 recorded blood losses was 350 mls ranging from 50 to 5,500 mls and thirty-five patients (11.4%) were transfused (250–1500 mls).

The mean duration of one-lung ventilation was 120 (40–540) minutes and this was approximately two-thirds of the total duration of operative mechanical ventilation. 56.8% (n = 168) of anaesthetists used volume-controlled ventilation as opposed to pressure controlled ventilation. However, there was no noteworthy difference in the percentage of patients ventilated with plateau inspiratory pressures ≥ 25 cm H_2_O between the two modes.

Data on pain relief of 306 patients demonstrated that thoracic epidural was the most popular pain control technique (61.1%), followed by paravertebral block (31%). In a few cases paravertebral was combined with intrathecal morphine (1.3%). Other techniques included intrathecal morphine (1.6%), patient controlled analgesia (4.9%) and intermittent injections or infusions of opioids (1.3%). At the end of surgery, 95.2% (n = 275) of patients had intercostal chest drains inserted.

### Post-operative management

Data on the post-operative management location of 308 patients (98.7%) were available. The majority were post-operatively managed on critical care areas (88.1%) although 10.6% were post-operatively managed on surgical wards (11.8% data unknown). Of the 44 ICU admissions, 27 were unplanned. The median fluid balance at 24 hours was +16.2 ml/kg (-42.3 to 87.6 ml/kg).

### Outcome following pneumonectomy

A total of 133 major complications occurred in 99 of 312 patients (31.7%). Table 2 shows the frequency of the individual components of the major complications.

**Table 2:** Frequency of individual components of the major complications

**Major complications**	**n (%) of patients**
Treated cardiac arrhythmias	62 (19.9)
Unplanned ICU admissions	28 (9.0)
30 day mortality	17 (5.4)
Further surgery	15 (4.8)
Inotrope usage	11 (3.5)

Developing a major complication resulted in an increased median length of hospital stay of an additional 2 days (8 (4–23) days vs. 10 (1–63) days).

The overall 30 day and in-hospital mortality rates after pneumonectomy were 5.4% (n = 17) and 4.8% (n = 15), respectively. Respiratory causes accounted for 58.8% of 30 day mortality (pneumonia, n = 3; pneumonia following broncho-pleural fistula, n = 2; acute respiratory distress syndrome, n = 3; pulmonary aspiration, n = 1; and respiratory failure, n = 1). Major haemorrhage accounted for 23.5% (n = 4) and myocardial infarction for 17.6% (n = 3).

### Risk factors for major post-operative complications

In univariate analysis, preoperative oxygen saturation less than or equal to 94% and FEV_1 _were associated with major complications, (Table 3).

**Table 3:** Risk factors associated with major complications in univariate analysis

**Risk factors**	**Numbers**	**Odds ratio (95%CI)**
Age	312	1.07 (1.03, 1.10; P < 0.001)
ASA: ≥ P3 vs < P3	137, 173	1.9 (1.1, 3.0; P = 0.01)
DLCO mmol/min/kPa	153	0.76 (0.62, 0.92; P = 0.006)
Epidural vs paravertebral	187,90	1.8 (1.0,3.2; P = 0.05)
FEV_1 _L	309	0.67 (0.45, 1.00; P = 0.05)
FEV_1 _% predicted	308	1.00 (0.98,1.01; P = 0.7)
ppo FEV_1 _% predicted	306	0.99 (0.96,1.01; P = 0.4)
DLCO % predicted	154	0.99 (0.97,1.01; P = 0.13)
ppo DLCO % predicted	162	0.97 (0.93,1.01; P = 0.11)
Preop. O2 saturation on air: ≤ 94 vs > 94%	26, 235	2.3 (1.0, 5.2; P = 0.04)
One lung ventilation: (hours)	294	1.09 (0.99, 1.20; P = 0.1)
Plateau pressure during one lung ventilation: ≥ 25 vs < 25 cmH_2_O	254	1.5 (0.8, 2.7; P = 0.2)
Right vs left operations	110, 199	1.5 (0.9, 2.5; P = 0.08)
Fluid positive balance	272,19	0.9 (0.3, 2.5; P = 0.9)
Planned vs Converted	208,102	1.3 (0.8,2.2; P = 0.3)
Gender: Male vs Female	209,101	1.2 (0.7,2.0; P = 0.4)
Stump closure: staples vs sutures	249,36	1.1 (0.5,2.3; P = 0.9)
Smoking history: yes vs no	286,25	1.2 (0.5,3.0; P = 0.7)
Neo-adjuvant therapy: yes vs no	33,270	0.5 (0.2,1.1; P = 0.1)

Stepwise multiple logistic regression analysis showed that advanced age, high ASA Physical Status and epidural analgesia were statistically significant associates of major complications at p < 0.05 (Table 4). In the roughly 50% of cases for which DLCO was recorded, low DLCO was also strongly associated.

**Table 4:** Risk factors associated with major complications in multivariate analysis

	**Multivariate (n = 275)**	**DLCO included (n = 141)**
**Risk factors**	**Odds ratio (95%CI)**	**Odds ratio (95%CI)**
Age	1.07 (1.04, 1.11; P = 0.001)	1.07 (1.02, 1.11; P = 0.004)
ASA: ≥ P3 vs < P3	1.7 (1.0, 2.9; P = 0.05)	
DLCO mmol/min/kPa		0.78 (0.64, 0.95; P = 0.02)
Epidural vs paravertebral	2.2 (1.1, 3.8; P = 0.02)	

**Figure 1:**
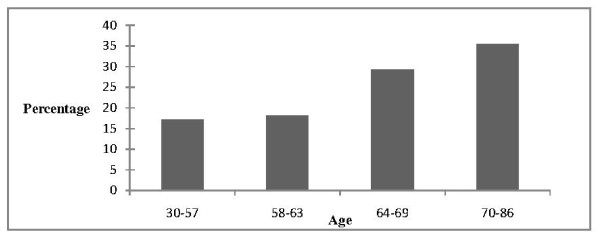


The risk of developing a major complication increased throughout the age ranges in the study, (Figure 1).

## Discussion

### Principal Findings

The short term risks of pneumonectomy as demonstrated by this UK study were; in-hospital and 30-day mortality rates of 4.8% and 5.4% respectively, and rates of major complications of 31.7%.

This rate of major complications is in agreement with published data (range 17–47%).[1,2,4,6,12] Treated cardiac arrhythmias were the commonest major complication affecting 19.9% (n = 62) of patients. This is also consistent with published data (range 16.5–26.1%).[1,2,4,6].

Increasing age, higher ASA Physical Status, preoperative reduced DLCO values and epidural analgesia were all found to contribute to risk factors collectively associated with an increased rate of major complications.

### Strengths and limitations

Previous studies on pneumonectomy outcome have often used small numbers of pneumonectomies, [1-5] been retrospective,[6,7,9,13-15] or carried out over prolonged periods of time .[6-10] Consequently there have been conflicting findings concerning the important risk factors for poor outcomes following a pneumonectomy.

The main strength of this study is that it is the largest prospective investigation into morbidity and mortality following elective pneumonectomy for cancer, and therefore the findings are representative of current UK practice. The Society of Cardiothoracic Surgeons of Great Britain returns for 2005 – 2006 recorded 416 pneumonectomies for primary lung cancer, but this number would include completion or extended pneumonectomies not relevant to our study.[11] Our data capture of 312 pneumonectomies represents at least 75% of the case load performed in the UK in a calendar year. Another strength of this study is that it was carried out over one calendar year to minimise variations in clinical practice over time.

The main limitation of this study is due to its observational nature and therefore observations on risk factors for poor outcome may be subject to unknown bias of various kinds. However because the data represent such a large portion of UK practice in this area, it is of interest to see the factors apparently most important though the results must be interpreted with some caution.

### Interpretation of risk factors for major complications in the context of previous studies

#### 1) Age

Based on Grade B evidence, the British Thoracic Society (BTS) guidelines state that age should be a factor in deciding on suitability for pneumonectomy and that mortality should be less than 8%.[16] The American College of Cardiology and American Heart Association (ACC/AHA) practice guideline update on perioperative cardiovascular evaluation for noncardiac surgery 2002 indicates that patients who are 70 years or older undergoing major thoracic surgery carry a particularly high risk for perioperative cardiac morbidity.[17]

The median age of our study population was 63 years which is consistent with the median age (58–65 years) in current publications on patients undergoing elective pneumonectomy for lung cancer.[1,2,4,6,9,12,18,19] The incidence of lung cancer peaks in people in their late 70s and is 3.3-fold higher in the 70–80 year than the 60–70 year age group,[10,20] which indicates that age is one factor used worldwide to minimize poor outcome following lung surgery. A number of studies demonstrate that advanced age is a strong predictor of morbidity and/or mortality following lung resection,[10,15,21-23] and pneumonectomy[1,6,18]. A small amount of evidence supports no association between advanced age and morbidity.[2,14] Our study not only shows a significant difference in age between the groups suffering or not-suffering a major complication including death, but also shows the risk increases in a stepwise manner across the quartiles. Patients older than 62 years had up to a 5-fold increase in the rate of major complications. This quantitative analysis of the relationship has a notable implication on future selection of patients with lung cancer for pneumonectomy. In the United States, it is conceivable that the number of persons older than 65 years undergoing thoracic and other major non-cardiac surgery will increase from the current 6 million to nearly 12 million per year.[17] Hence, limiting an effective surgical procedure to the proven low risk age group cannot be the only way to improve post-operative outcomes.

#### 2) ASA Physical Status

This study has shown that an ASA Physical Status of more than or equal to P3 is associated with poor outcome. Despite its widespread clinical use, the value of the ASA Physical Status as a predictor for lung resection outcome has only been investigated in a couple of studies, which have shown it to predict both morbidity[24] and mortality.[9] Licker et al found it to be significant at predicting mortality in univariate analysis but not multivariate.[12]

Although the ASA Physical Status is a subjective assessment made by the Anaesthetist, it is based on many patient factors including co-morbidities and exercise capacity, which may explain why it is significant in predicting major complications.

#### 3) Lung function

The British Thoracic Society recommend that if the FEV_1 _is more than 2 litres, in the absence of interstitial lung disease or unexpected shortness of breath, further respiratory function tests are not required to assess suitability for pneumonectomy.[16] Our study found that the FEV_1 _had a marginally significant association with major complications in univariate but not multivariate analysis. However, we did not find any suggestion of association between the percent predicted FEV_1 _or the minimum post-operative percent predicted value (ppo) with major complications. The study median minimum post-operative percent predicted FEV_1 _was 39.3%, which indicates that the absence of an association was not due to high overall values. Perhaps the reason that our study failed to suggest an association was because patients with low ppo FEV_1 _values were then carefully selected for surgery as recommended by the British Thoracic Society guidelines[16]. Evidence from the literature is conflicting concerning the usefulness of FEV_1 _values as risk factors. Although FEV_1 _and its determinants have been found to predict morbidity or mortality in univariate analysis,[6,21,24,25] only a few studies have found it to contribute as predictor of morbidity or mortality in multivariate analysis.[5,26,27] Further studies have not found any association between FEV_1 _variables and morbidity or mortality.[1,2,4,12] This conflicting evidence is probably explained by variations in practice once a low FEV_1 _value is identified, in terms of further assessment and management. A limitation of this study is that the ppo FEV_1 _values were calculated without a ventilation/perfusion scan and termed minimum values in the text.

For the 49% of cases for which it was measured, the DLCO was a significant predictor of complications post-pneumonectomy but it was the raw DLCO measurement that was significant rather than the adjusted DLCO expressed as a percentage of predicted normal. Diffusing capacity declines with age, and age is one factor within the calculation of DLCO percent predicted normal. Elderly patients will, therefore, have low absolute DLCO values which will correct favourably as percent predicted normal for a patient of that age. This suggests that a critical amount of diffusion capacity is required to undergo pneumonectomy without complication. Diffusing capacity is not measured as routinely as spirometry prior to lung resection, a finding that Ferguson et al have challenged with studies showing that not only is it the best single predictor of complications, but also that it remains the best predictor for patients with normal spirometry.[21,28] However, Ferguson et al found that it was the post-operative predicted DLCO that was important and not the raw value. This difference may be because they studied all lung resections where as our study was limited to pneumonectomy. However our results support the view that diffusing capacity should be measured more frequently and the value used in the risk assessment to determine suitability for pneumonectomy.

#### 4) Fluid balance

Previous studies by Licker et al.[25] and Alam et al[29] have found a clear link between fluid therapy and incidence of post-lung resection acute lung injury (ALI). We found no such link between fluid therapy and major complications. The positive fluid balances in our group were small with the complication group having a median fluid balance of 20 ml/kg at 24 hours. Our results may be seen as confirming the 'beneficial' effect of thoracic anaesthetists keeping to current recommendations for fluid therapy post-pneumonectomy. However there were a number of wayward fluid balances reported and no obvious ill-effects from this in the small number of patients reported. This supports the contention that excessive fluid administration exacerbates hypoxemia in patients who develop ALI, but does not initiate the lung injury.

#### 5) Side of surgery

Most evidence supports increased complications and mortality following right sided lung resections[4,7,18,27,30,31] which is partially explained by an increased risk of broncho-pleural fistulae. However, others have found no significant difference.[2,6,12] This might be because of the limited power of smaller studies or the difficulty with analyzing data collected over many years. In our large study, the proportion of right-sided surgery was 35.3%, which might indicate that side of surgery is used to determine suitability for surgery. Our study found a higher complication rate (38%) with right-sided surgery than left (29%), which is only weakly significant (p = 0.08).

#### 6) Analgesic technique

Our study found that thoracic epidural was the most popular analgesic technique (61.1%) followed by paravertebral block (31%). Further, epidural analgesia was a significant associate of poor outcome in both univariate and multi-factor analysis. The possible explanation for this is that the unilateral nature of paravertebral blockade prevents hypotension and respiratory complications that can be associated with bilateral sympathetic blockade. Davies and Joshi et al recently published systemic and meta analysis reviews comparing the analgesic efficacy and side effects of paravertebral and epidural blockade for thoracotomy[32,33] which concluded that paravertebral blockade provided the same analgesic efficacy to epidural blockade but with fewer common side effects, most importantly less hypotension and pulmonary complications. This study provides additional evidence that epidural analgesia is associated with major complications, which can in themselves, lead to increased hospital length of stay.

### Clinical implications

This study has provided information on the short term risks associated with pneumonectomy in terms of mortality and major complication rates. The results from this study suggest that age should be central in the assessment of risk for a patient requiring pneumonectomy. The predictive nature of ASA Physical Status implies that anaesthetists accurately assimilate risk. DLCO was strongly associated with major complications, which suggests that DLCO should be measured more frequently, and used in the pre-operative risk assessment in pneumonectomy patients. Finally this study found epidural blockade was associated with major complications, which suggests that paravertebral may be the analgesic method of choice for pneumonectomy. However further research is needed to clarify this.

## Conclusion

The UK Pneumonectomy Outcome Study is the largest ever prospective study into mortality and morbidity following pneumonectomy for lung cancer surgery. The findings suggest that age is the best single predictor of morbidity following this surgery. Following multivariate analysis age, DLCO, ASA Physical Status and epidural analgesia were significant associates of major complications.

## Competing interests

The authors declare that they have no competing interests.

## Authors' contributions

EP was involved in analysing the data and drafted the manuscript. AP, GB and FG had the concept and designed and set up the study across the U.K. They also were involved in critical review of the manuscript. DC contributed to the design of the study and was chiefly responsible for the acquisition of data. EB and PD were involved in data analysis and interpretation. All authors read and approved the final manuscript.

## Supplementary Material

Additional file 1 Essential fields from the data collection sheet. Click here for file
