# Supplementary material for: UK pneumonectomy outcome study (UKPOS): a prospective observational study of pneumonectomy outcome
Source: J Cardiothorac Surg. 2009 Jul 30;4:41. doi: 10.1186/1749-8090-4-41 (PMC2726136; doi:10.1186/1749-8090-4-41)
Supplement: Additional file 1 — Essential fields from the data collection sheet. [file 1749-8090-4-41-S1.doc]

**ADDITIONAL FILE 1:**  **Essential fields from data collection sheet**

Centre code

Patient number

Date of surgery

Age of patient

Gender

Height (m)

Weight (kg)

BMI (kg/m^2^)

Exercise capacity (distance on flat in metres)

Alcohol intake (units per week)

Ever smoked (yes/no)

Pack year history (average number of cigarettes smoked per day divided by 20, multiplied by total number of years smoked)

Time since stopped smoking (weeks)

ASA physical status

Co-morbidities (Include all cardiac, respiratory, renal and vascular history and any other potentially relevant past history)

Pre-operative medication (Include steroids, NSAIDs, antifungals, leukotriene receptor antagonists, beta-2 agonists, and all ‘cardiac drugs’)

Lung cancer stage (pre-operative)

Cancer cell type

Adjuvant chemo- or radiotherapy (what drugs/ radiation/ dose/ regime /and when)

FEV^1^ (actual value in litres), FEV^1^ % (as percent of predicted value)

FEV^1^ / FVC (as percentage)

DLCO (actual value), DLCO % (as percentage of predicted normal)

KCO (actual value), KCO % (as percent of predicted normal)

Arterial line inserted

Central venous pressure line inserted

Mini-trach or tracheostomy inserted (if so when inserted and time in-situ)

Pressure or volume controlled ventilation (during surgery)

Fluids given during surgery (list fluid given in millimetres and type of fluid given)

Drugs given during surgery and during post-operative period (all non anaesthetic drugs, e.g. diuretics, inotropes, antibiotics not normally taken by patient, received during the first 48 hours post-operatively and for entire length of stay on ICU/HDU)

Blood loss during surgery

Planned or converted

Duration of intermittent positive pressure ventilation (in minutes)

Duration of one-lung ventilation (in minutes from initiation to completion of surgery)

Duration of surgery (in minutes from skin incision to final suture)

Initial post-operative location

Unplanned ICU admission

ICU length of stay (in days)

Post-operative pain relief (state which drugs, regime and route)

Fluids given after surgery in first 24 hours

Output (fluid output during surgery up to 24 hours- include urine output, chest drain output and any other losses)

Fluid balance (fluid balance at 24 hours post-operatively, give figure in millimetres and whether positive or negative)

Post-operative complications (list organ systems and exact complications for respiratory, cardiovascular, renal, hepatic systems, sepsis, multi-organ failure)

Post-pneumonectomy pulmonary oedema (diagnosed as hypoxia PF ratio < 40, bilateral infiltrates on chest x-ray, PAP < 18mmHg when measured, cardiac index > 3 when PiCCO or oesophageal Doppler used, PEEP requirement ≥ 7.5mmHg)

Survived to discharge

Length of stay in hospital (to death or discharge in days)

Cause of death (from death certificate or post mortem)
